# Splay Toe after Freiberg-Köhler's Osteonecrosis: A Case Report of a Successful Operative Treatment in a Rare Multiplanar Foot Deformity

**DOI:** 10.1155/2020/8830166

**Published:** 2020-11-19

**Authors:** Martin Riegger, Marco Guidi, Giuseppe Filardo, Christian Candrian

**Affiliations:** ^1^Department of Orthopedic and Trauma Surgery, Ospedale S. Giovanni di Bellinzona e valli, Ospedale Regionale di Lugano, Via Ospedale, CH-6500 Bellinzona, Switzerland; ^2^Department of Plastic Surgery and Hand Surgery, Universitätsspital Zürich, Rämistrasse 100, Zürich, CH 8091, Switzerland; ^3^Istituto Ortopedico Rizzoli, Università degli Studi di Bologna, Scuola di Medicina e Chirurgia, Italy; ^4^Department of Orthopedic and Trauma Surgery, Ospedale Regionale di Lugano, Via Tesserete 46, Lugano, Ticino, CH 6900, Switzerland

## Abstract

“Splay toe” is a rare deformity of the forefoot and often causes the occurrence of metatarsalgia and dysfunction while walking or weight bearing. Since it involves a deviation in the sagittal and transversal planes, often combined with a malrotation, surgical correction can be challenging. We describe a case of splay toe deformity in the forefoot causing metatarsalgia in a 62-year-old female patient with a former avascular osteonecrosis of the 2 metatarsal head Smillie stage V of Freiberg-Köhler's disease causing a splay toe between the 2^nd^ and the 3^rd^ rays. There are only few reports in the literature, and a clear treatment strategy has not been defined, yet, although, it has been described that most of these patients are operated more than once. In the presented case, we performed a successful treatment by a combined surgical technique consisting in modified Weil's osteotomy and the transfer of the extensor brevis tendon. We sustain that for correction of a multiplanar deformity of lesser toe deformities osseous correction as well as tendon transfer lead to successful therapy.

## 1. Introduction

In the literature, there are many reports of lesser toe deformities such as hammer toe, mallet toe, and claw toe. Their right treatment is still debated and ranges from conservative therapy to surgery [1]. Surgical treatment can be performed with a conventional open technique or through minimal invasive surgery [2]. Among surgical options, both soft tissue corrections, such as extensor brevis tendon transfer, as well as osseous correction, like Weil's osteotomy, have been described [3–5]. Among these deformities, particularly challenging and debated is the treatment of the Splay toe deformity, a rare presentation that involves the lesser toes, which has been documented only by a few reports. We present a case of clinical, radiological, and functional outcome of a multiplanar deformity of the forefoot causing a splay toe due to Freiberg-Köhler's osteonecrosis.

## 2. Case

**Figure 1:**
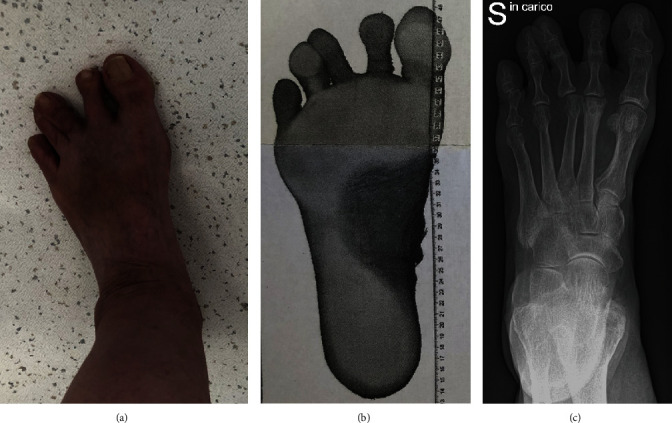
(a) Splay toe between 2^nd^ and 3^rd^ toe. (b) Dynamic analysis of the foot while weight bearing. (c) Plane X-ray of the foot showing Smillie stage V necrosis of the 2^nd^ metatarsal head causing the splay toe deformity with a relatively long 3^rd^ metatarsus.

**Figure 2:**
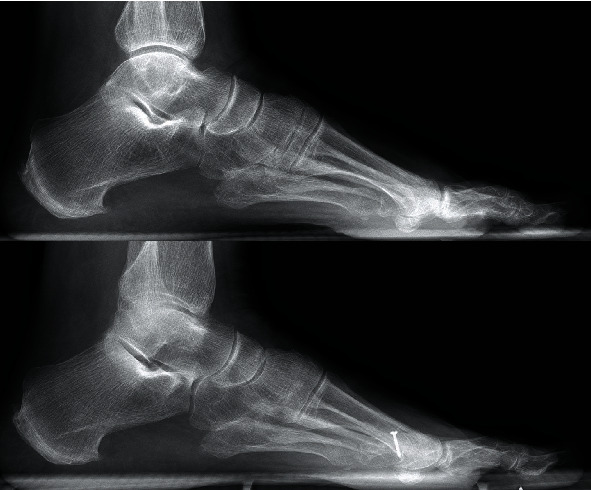
Lateral radiograph prior surgery and at three months follow-up.

A 62-year-old female presented a metatarsalgia of the right mid- and forefoot with a splay toe two-to-three (Figure 1(a)). The deformity had been caused by an avascular osteonecrosis of the second metatarsal head (Freiberg-Köhler) developing a relatively short 2^nd^ metatarsus causing deviation of the third toe laterally. We excluded rheumatic arthritis (RA). Walking analysis showed worsening symptoms of pain and deformity while weight bearing (Figure 1(b)). Normal walking without severe metatarsalgia and conflict between 3^rd^ and 4^th^ toe was not possible. Conservative treatment with redressing bandages and rigid plantar soles failed. X-ray in the ap view showed the deformity weight bared (Figure 1(c)).  Figure 2(a) showed the deformity in the lateral view.

**Figure 3:**
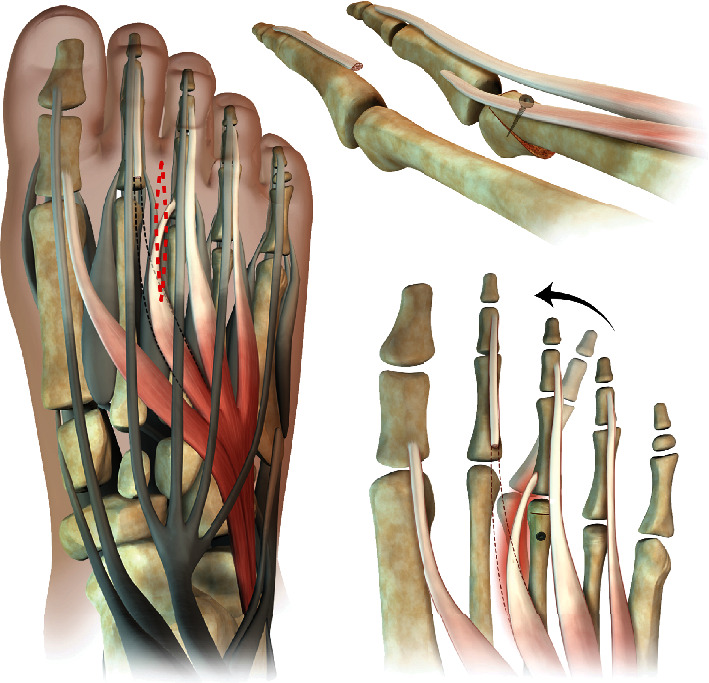
Schematic chart of the surgical procedure for splay toe correction: Modified Weil's osteotomy with a lateral rotation of the metatarsal head. Extensor brevis tendon transfer from the second ray to the base of the proximal phalanx of the third toe.

**Figure 4:**
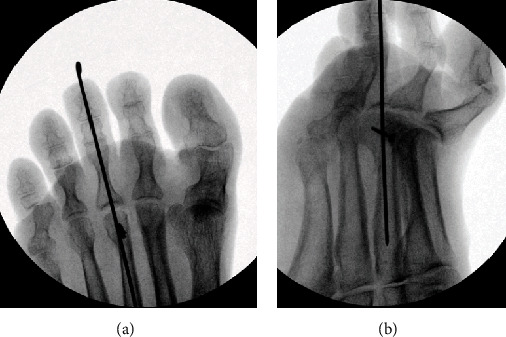
Intraoperative X-ray after realignment performing Weil's osteotomy, extensor brevis transfer, and temporary arthrodesis of the second toe.

**Figure 5:**
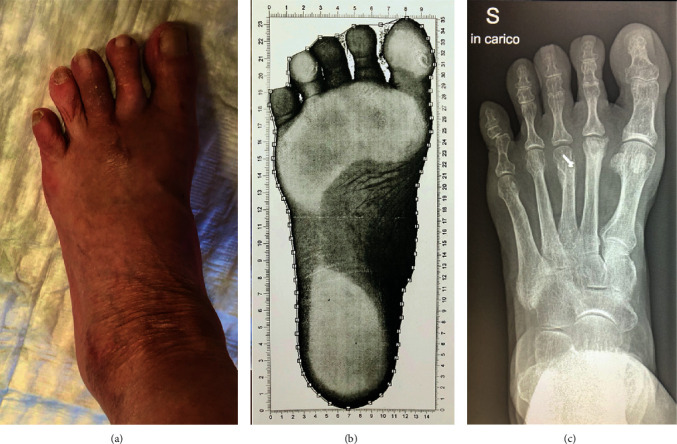
Results at the 3-month follow-up after removing the K-wire, with (a) photograph while weight bearing, (b) walking analysis, and (c) plane X-ray.

A surgical approach with a modified Weil's osteotomy of the 3^rd^ metatarsus was performed. The modification of the classic Weil's osteotomy consisted in a lateral rotation of the metatarsal head to achieve correction due to both shortening and rotation in the transversal plane. Transfer of the 2^nd^ extensor brevis tendon to the base of the proximal phalanx of the 3^rd^ toe was performed. The operation is shown on the schematic chart in Figure 3. During the operation, integrity of plantar plate and collateral ligaments was tested and assured. To allow soft tissue healing, a K-wire was inserted in a temporary arthrodesis to keep the axis with partial weight bearing (Figures 4(a) and 4(b)). K-wire removal was performed after 6 weeks with subsequent return to full weight bearing (Figure 5(a)). Technical walking analysis was performed for the functional outcome (Figure 5(b)). X-rays were taken to evaluate radiological outcome in ap view (Figure 5(c)) and lateral view (Figure 2(b)). Metatarsalgia was measured with the VAS (Visual Analogue Scale) showing a reduction of the pain level from 7 prior surgery to 0 after three months. The AOFAS lesser toes score was used to measure the overall outcome. It showed improvement from 38 before surgery to 95 after rehabilitation time of three months. There was no remaining rigidity reported.

## 3. Discussion

Treatment of deformities such as claw toe, mallet toe, hammer toe, and even crossover toe has been discussed widely in the literature [1, 6–12]. Although many therapeutic options have been described in the last two decades, a “splay toes deformity” can be a surgical challenge even to the most experienced foot and ankle surgeon [6, 12]. This is probably due to the complex multiplanar component of this pathology. Whereas mallet toe or hammer toe develops in one plane due to a tendon disorder, splay toe derives from a problem in at least two planes (axial and transversal) often comprising of an additional rotational deviation. For surgical success to be achieved, all planar defects must be addressed.

In our patient case, the relatively short second metatarsal was responsible for the lateral deviation of the third toe, which provoked a caudal rotation, causing a partial overlapping of the fourth toe. Furthermore, the third metatarsal deviated medially, creating tension on the second to and subsequent medial deviation. The origin of all these anatomical deformities arose from the shortening of the second metatarsal due to a Smillie stage V osteonecrosis [13, 14]. Other causes for splay toe development such as primary rheumatoid arthritis were excluded prior to surgical intervention. To measure the quality of our result clinically, the VAS (Visual Analogue Scale) was used as well as the AOFAS score for lesser toes [15–17].

For successful corrective treatment, the alignment in length, plane, and rotation had to be restored. To this reason, we chose to perform a Weil's osteotomy to reestablish the length. The osteotomy was modified as shown on the schematic view (Figure 3) rotating the head laterally in order to adjust the positioning within the transverse plane and permit the transfer of the extensor brevis tendon to stabilize the rotation forces, while maintaining positioning in the frontal plane.

A temporary metatarso-transphalangeal arthrodesis for 6 weeks had been done for soft tissue and bone consolidation. The time of 6 weeks did not have any impact on range of motion of the lesser toes as shown in the AOFAS score of lesser toes who improved in all qualities. At 3 months follow-up, the radiological and clinical findings demonstrated a successful outcome of this combined treatment strategy. It showed that the correction of the deformity remained stable without any loss in the range of motion.

## 4. Conclusion

The splay toe deformity is a rare pathology with a complex management. It is a multidirectional deformity in the sagittal and transversal plane together with a rotational component. In this rare case, caused by Freiberg-Köhler's disease, surgical management and planar reconstruction led to satisfactory result. However, further studies are needed to define a clear therapeutic algorithm and surgical consensus for the treatment of these challenging splay toe deformities.

## Data Availability

All data is available by the author.
